# *Nigella sativa* callus treated with sodium azide exhibit augmented antioxidant activity and DNA damage inhibition

**DOI:** 10.1038/s41598-021-93370-x

**Published:** 2021-07-06

**Authors:** Mohammed Shariq Iqbal, Zahra Iqbal, Abeer Hashem, Al-Bandari Fahad Al-Arjani, Elsayed Fathi Abd-Allah, Asif Jafri, Shamim Akhtar Ansari, Mohammad Israil Ansari

**Affiliations:** 1 Amity Institute of Biotechnology, Amity University Uttar Pradesh, Lucknow Campus, Lucknow, 226 028 India; 2 Molecular Crop Research Unit, Department of Biochemistry, Chulalongkorn University, Bangkok, 10330 Thailand; 3 Botany and Microbiology Department, College of Science, King Saud University, P.O. Box. 2460, Riyadh, 11451 Saudi Arabia; 4 Plant Production Department, College of Food and Agricultural Sciences, King Saud University, P.O. Box. 2460, Riyadh, 11451 Saudi Arabia; 5 Molecular Endocrinology Lab, Department of Zoology, University of Lucknow, Lucknow, 226 007 India; 6 Institute of Forest Productivity, Ranchi, 835303 India; 7 Department of Botany, University of Lucknow, Lucknow, 226 007 India

**Keywords:** Biochemistry, Biotechnology, Plant sciences

## Abstract

*Nigella sativa* L. (NS) is an herbaceous plant, possessing phytochemicals of therapeutic importance. Thymoquinone is one of the active phytochemicals of NS that confers noteworthy antioxidant properties. Sodium azide, an agent of abiotic stress, can modulates antioxidant system in plants. In the present investigation, sodium azide (0, 5 µM, 10 µM, 20 µM, 50 µM, 100 µM and 200 µM) doses administered to the in vitro NS callus cultures for production/modification of secondary metabolites with augmented activity. 200 µM sodium azide treated NS callus exhibited maximum peroxidase activity (1.286 ± 0.101 nanokatal mg^−1^ protein) and polyphenol oxidase activity (1.590 ± 0.110 nanokatal mg^−1^ protein), while 100 µM sodium azide treated NS callus for optimum catalase activity (1.250 ± 0.105 nanokatal mg^−1^ protein). Further, 200 µM sodium azide treated NS callus obtained significantly the highest phenolics (3.666 ± 0.475 mg g^−1^ callus fresh weight), 20 µM sodium azide treated NS callus, the highest flavonoids (1.308 ± 0.082 mg g^−1^ callus fresh weight) and 100 µM sodium azide treated NS callus, the highest carotenes (1.273 ± 0.066 mg g^−1^ callus fresh weight). However, NS callus exhibited a decrease in thymoquinone yield/content vis-à-vis possible emergence of its analog with 5.3 min retention time and an increase in antioxidant property. Treatment with 200 µM sodium azide registered significantly the lowest percent yield of callus extract (4.6 ± 0.36 mg g^−1^ callus fresh weight) and thymoquinone yield (16.65 ± 2.52 µg g^−1^ callus fresh weight) and content (0.36 ± 0.07 mg g^−1^ callus dry weight) and the highest antioxidant activity (3.873 ± 0.402%), signifying a negative correlation of the former with the latter. DNA damage inhibition (24.3 ± 1.7%) was recorded significantly maximum at 200 µM sodium azide treatment. Sodium azide treated callus also recorded emergence of a new peak at 5.3 min retention time (possibly an analog of thymoquinone with augmented antioxidant activity) whose area exhibits significantly negative correlation with callus extract yield and thymoquinone yield/content and positive correlation with antioxidant activity and in vitro DNA damage inhibition. Thus, sodium azide treatment to NS callus confers possible production of secondary metabolites or thymoquinone analog (s) responsible for elevated antioxidant property and inhibition to DNA damage. The formation of potent antioxidants through sodium azide treatment to NS could be worthy for nutraceutical and pharmaceutical industries.

## Introduction

*Nigella sativa* L. (NS) a medicinal herb, commonly known as black seed/cumin, belongs to the family Ranunculaceae. NS is a native plant of Mediterranean and south Asian regions mainly India, Pakistan and Sri Lanka^1^. Various studies encircling the medicinal properties of NS highlighted its promising effects such as anti-cancer, anti-inflammatory, anti-parasitic, anti-analgesic, anti-microbial, anti-stress, antioxidant and anti-asthmatic^2–4^. A recent study showed its protective effect against diazinon cardio toxicity in rat model^5^. These properties of NS are mainly because of active phytochemicals present in it^6^. Phytochemicals are basically secondary metabolites and extensively utilized in herbal medicine, dietary regimens, fragrance industry, flavoring industry, and for preservation purposes^7–9^. Thus, secondary metabolites owing antioxidative potential and various imperative biological properties possess vast applications.

NS seed oil contains various secondary metabolites viz*.,* p-cymene monoterpenes, β-pinene, α-thujene, γ-terpinene, α-pinene, nigellone, caracole, thymoquinone and thymol^10^. Tiruppur et al.^11^ have reported that NS possesses phenolic and quinone compounds. Of these, thymoquinone is one of the most predominant and active phytochemicals of NS seed oil^12^. Hence, numerous studies have been performed on pharmacological importance of thymoquinone^13,14^ that has been associated with NS anti-carcinogenic activity^15^**.** On similar lines, a previous study reports the cytotoxic effect of thymoquinone on keratinocyte cells^16^. Moreover, thymoquinone also possess anti-tumor and antiproliferative activity, preventing progression of tumors via angiogenesis and metastasis^17^. On contrary to the traditional treatments like radio or chemotherapy, thymoquinone can destroy cancerous cells by specific targeted mechanisms^18,19^. Several in vivo and in vitro studies on thymoquinone antitumor activity have revealed significant apoptotic effects^20^. However, other studies revealed thymoquinone as of low potential therapeutic secondary metabolite, because of its deprived effectiveness and bio-availability^21,22^. Additionally, low water solubility and cytotoxic effects at higher dosage also constraints its usage as a therapeutic biomolecule. However, the advent of high-throughput omics technology, allowing quantitative measurements of putative genes responsible in the biosynthetic pathways of key secondary metabolites, has circumvented the limitations associated with the application of plant based secondary metabolites in drug discovery^23^. Recently, SWATH-MS (sequential window acquisition of all theoretical fragment ion-spectramass spectrometry) technique has been extended to plant systems for quantifying proteome dynamics^24^. As genetic information of NS is limited, proteogenomics technique would be helpful in revealing genomic annotation of NS, as proteogenomics (combination of genomics, proteomics, and transcriptomics) has considerably improved genome annotation useful for elucidating homology information of phylogenetic groups^25,26^. All these robust techniques are profusely being applied to plant systems for the augmented production of secondary metabolites that are beneficial for nutraceutical and pharmaceutical industry.

Furthermore, plants alter/elevate their phytochemicals/secondary metabolites to fight against the oxidative stress if they are subjected to stress or various adverse conditions^27^. Generally, the foremost effect of several environmental stresses, including salt stress generates reactive oxygen/nitrogen species (ROS/RNS) and enhances oxidative stress load in plants^28^. These ROS/RNS vigorously interact with several cellular components, leading to substantial injury to various cellular organelles^29^. In order to combat these ROS/RNS, plants produce antioxidants as a defense mechanism to scavenge ROS/RNS^30^. The biosynthesis of polyphenol in plants is generally accumulated due to the stimulation of biotic/abiotic stress which plays a vital role in combating ROS/RNS^31^. Under stress conditions, the plants produce various enzymatic (peroxidase, catalase, polyphenol oxidase, superoxide dismutase, etc.) and non-enzymatic (phenolic compounds, flavonoids, carotene, etc.) antioxidants^31^. Thus, antioxidants serve a boost to plant tolerance against stress condition.

Therefore, in order to elicit secondary metabolites (antioxidants), in vitro technique like plant tissue culture is used to obtain amplified quantities in a time judicious manner. Several plant tissue culture methods have been comprehensively investigated in order to increase the fabrication of secondary metabolites produced by plant^32^. Several biotic or abiotic factors through elicitation technique in plant tissue culture are commonly used for obtaining high fabrication yield of desired secondary metabolites. Elicitors in callus cultures are generally signaling molecules inducing the signal transduction cascade. This eventually results in expression of related gene responsible for synthesis of secondary metabolites^33^. In a study by Garg et al.^34^ where transcriptome changes due to stress caused by sodium azide treatment have been reported; and further because of such changes, the role of RGG-motif proteins was studied. Hernández et al.^35^ administered sodium azide and mannitol to induce stress condition in maize germination that led us to study the effect of sodium azide on NS using tissue culture technique. Thus, the present investigation aimed at testing graded doses of sodium azide to NS callus culture. This could help to obtain high yields of efficient antioxidants, including thymoquinone analog(s)^36^ that could be employed to test their potential for DNA damage inhibition. Moreover, if more potent antioxidants could be produced by such a technique, it would be beneficial for nutraceutical and pharmaceutical industry for herbal drug development using NS.

## Materials and methods

All plant studies (*Nigella sativa L.)* were carried out in accordance with relevant institutional, national or international guidelines and regulation.

### Explant preparation

The seeds of NS were procured from National Research Centre on Seed Spices (NRCSS), Rajasthan, India and designated as Ajmer Nigella 1 (AN1). The callus formation and extract preparation were done according to the published reproducible protocol^37^. NS seeds were washed for two min with detergent solution and then placed under running tap water for 1 h. The seeds were then sterilized with 0.1% mercuric chloride (Extra pure; HiMedia, GRM1067) for 30 s, followed by additional five washings with sterile water under laminar air flow (Laczene Biosciences, India). Finally, the seeds were rinsed with 70% ethanol (AR grade, 99.9% purity, Merck, India) for 30 s followed by five consecutive washes with sterile water. The sterile seeds were then transferred onto agar-solidified Murashige and Skoog Medium (MS media) (HiMedia, PT100)^38^. The culture bottles were kept in dark for about six days until germination. Post 10 days of germination, seedlings were collected on sterile culture plates. For callus formation, the seedlings were trimmed into 2 cm length from epicotyl region and transferred on agar-solidified MS media containing 1 mg 2, 4-D (2,4-Dichlorophenoxyacetic acid) L^−1^ (HiMedia, PCT0825). Four week-old calli were then transferred onto MS culture media supplemented with sodium azide (AR grade; HiMedia, GRM1038), previously optimized, so that it should not inhibit the callus biomass growth as reported^39,40^ at concentrations of 0 µM (control), 5 µM, 10 µM, 20 µM, 50 µM, 100 µM and 200 µM*.* Each treatment was performed in triplicate. The culture flasks were kept under cool fluorescent lamps at 150 lx of illuminance, ambient temperature of 25 ± 2 °C, and a photoperiod of 16 h day and 8 h night to yield healthy calli.

### Isolation of protein and estimation of different enzymatic antioxidants

#### Protein isolation and estimation

Total protein was isolated according to the protocol with some modifications^41^. 0.5 g of NS callus tissue was homogenized in 5 ml extraction buffer containing 50 mM (pH 6.8) tris HCl (AR grade; HiMedia, GRM613), 200 mM NaCl (AR grade; HiMedia, GRM853), 20–200 mM β-mercaptoethanol (AR grade; HiMedia, BM041) and incubated at 4 °C for 20 min. The homogenate was spun at 12,000 rpm for 15 min at 4 °C, using high spin cooling centrifuge (REMI, India C-24BL). Supernatant was used for total protein estimation on spectrophotometer (UV–Vis 1800 Shimadzu)^42^.

#### Peroxidase

Peroxidase activity assay was carried out following the previously published protocol^43^. The reaction mixture consisted of 50 mM Tris HCl (pH 6.8), 3 mM H_2_O_2_ (AR grade; HiMedia, GRM2444), 3 mM guaiacol (Extra pure; HiMedia, RM1118) (ε_470nm_ = 2.66 × 104 M^−1^ cm^−1^) and 100 µL of NS callus extract (10 mg mL^−1^). The activity was taken at 470 nm and increase in absorbance was noted using spectrophotometer (UV–Vis 1800 Shimadzu). Activity was measured in triplicates and the mean activity (± standard deviation) was calculated.

#### Polyphenol oxidase

Spectrophotometric activity of polyphenol oxidase was determined according to the protocol^44^. The reaction mixture consisted of 3.0 mM catechol (AR grade; HiMedia, GRM6782), 3.0 mM H_2_O_2_ and 50 mM Tris HCl (pH 6.8) and 100 µL of NS callus extract (10 mg mL^−1^) and measured at 420 nm. The molar extinction coefficient of catechol is (ε_420nm_ = 3450 M^−1^ cm^−1^). An increase in absorbance was noted using spectrophotometer (UV–Vis 1800 Shimadzu). Activity was measured in triplicates and the mean activity (± standard deviation) was calculated.

#### Catalase

Catalase activity was determined according to the protocol^45^. The reaction mixture consisted of 0.1% H_2_O_2_ in 50 mM Tris HCl (pH 6.8) and 100 µL of NS callus extract (10 mg mL^−1^). The method directly measured decrease in absorbance at 240 nm (ε_240nm_ = 43.6 M^−1^ cm^−1^) using spectrophotometer (UV–Vis 1800 Shimadzu), due to H_2_O_2_ consumption by catalase. Activity was measured in triplicates and the mean activity (± standard deviation) was calculated.

### Estimation of non-enzymatic antioxidants

#### Total phenolics

Total phenolic was estimated according to the method as mentioned^46^. 1.0 g of NS callus tissue weight was homogenized in 10 mL of 70% ethanol. Whatman no. 1 filter paper was used to filter the extract and stored at 4 °C for further use. The extract (0.5 mL) was mixed with Folin–Ciocalteu reagent (LR grade; HiMedia, RM10822) (previously diluted 1:10) yielding pale blue color; for neutralization 4 mL sodium carbonate (7.5%, w/v) (AR grade; HiMedia, GRM851) was added. To complete the reaction, it was incubated for 30 min at room temperature, followed by spectrophotometric measurement of absorbance at 765 nm. Gallic acid (GA) (AR grade; HiMedia, GRM233) was used as standard (Supplementary Figure S1).

#### Total flavonoids

Total flavonoid was extracted by the standard method^47^. 1.0 g of NS callus tissue was submerged in 4 mL of 80% ethanol for overnight and then homogenized using mortar pestle. The extract was filtered twice using Whatman no. 1 filter paper. Filtrate was collected in fresh tubes and stored at − 20 °C until further analysis. The ethanolic extract was taken and kept at room temperature, vortexed, and diluted to 1:10 with 80% ethanol, producing light yellow color. Absorbance was taken at 362 nm using spectrophotometer. Flavonoid quantification was done by using standard Quercetin (QE) (Sisco research laboratories Pvt. Ltd. (SRL), 71923) as standard (Supplementary Figure S2).

#### Total carotene

Carotene content was estimated according to the method^48^. NS callus (0.5 g) was homogenized in 5 mL of 100% acetone (LR grade; HiMedia, AS024). Whatman No.1 filter paper was used for filtration followed by centrifugation for 10 min at 2500 rpm. The supernatant was collected and the absorbance was taken at 662 nm (for chlorophyll A), 646 nm (for chlorophyll B) and 470 nm (for total carotene) using spectrophotometer and calculated according to the formulae as mentioned below^49^.

$${\text{Chlorophyll a}} = 11.75{\text{ A}}_{{662}} - 2.350{\text{ A}}_{{645}}$$

$${\text{Cholorophyll b}} = 18.61{\text{ A}}_{{645}} - 3.960{\text{ A}}_{{662}}$$

$${\text{Total carotene}} = 1000{\text{ A}}_{{470}} - 2.270{\text{ Ca}} - 81.4{\text{ Cb}}/227$$

$${\text{Ca}} = {\text{Chlorophyll a}},{\text{Cb}} = {\text{Chlorophyll b}}$$

### Extract preparation for RP-HPLC

Varying concentrations from 0 to 200 µM sodium azide treated healthy NS calli from each flask were used to prepare methanolic extracts (2.5 g NS callus in 10 ml methanol)^50^. The obtained methanolic extracts were analyzed on RP-HPLC. Filtered and dried NS callus extract (NSE) (previously weighed) were dissolved in methanol (HPLC-grade, Merck, India, CAS no 67-56-1) and then diluted to 1 mg mL^−1^ for stock solution; which was then filtered using 0.22 μm syringe filter (MCE hydrophilic membrane; HiMedia, SF2) for further analysis.

### Spectrum scanning analysis

Standard thymoquinone (98.0% purity; Cat. # 490915, Pubchem ID 24856596), Sigma-Aldrich, was used to determine the maximum absorbance and content in NS callus extract (Supplementary Figure S3A). Spectrum scanning analysis (UV–Vis 1800 Shimadzu) was done for absorbance in the designed experiments^51^. The standard thymoquinone spectrum scanning was compared with the NSE spectrum scanning for the preliminary identification of thymoquinone.

### RP-HPLC (reverse phase-high performance liquid chromatography) analysis

Ultra-Fast Liquid Chromatography (UFLC-Shimadzu), was performed for the RP-HPLC investigation^50,52^. HPLC with pump Model LC-20AD, and UV–Vis detector Model SPD-20A with C-18 column (250 mm × 4.6 mm) (Shiseido, Japan) was used. Acquisition of data was executed using Lab Solution Lite software (Shimadzu Corporation Kyoto, Japan). Standard pure thymoquinone (99.0% purity), Sigma-Aldrich, HPLC-grade methanol, Merk, 106007, HPLC-grade water, Merk, 115333, and Lab Solution Lite software—Shimadzu was used for analysis of the data.

The mobile phase consisted of methanol: water (70:30 v/v), with 1.0 mL min^−1^ flow rate and absorbance at 254 nm, at an ambient temperature of 25–30 °C. A retention time of 7.376 min was obtained. The mobile phase i.e., sample/solvent was filtered through 0.22 μm syringe filters and degassed with the help of bath sonicator. Standard thymoquinone stock solution of 1 mg mL^−1^ concentration was prepared in methanol while taking caution for stability (sensitive to light and heat). 0.0312 to 2.0 μg mL^−1^ of concentrations of thymoquinone stock solution was used to plot calibration curve (Supplementary Figure S3B). For the confirmation of peak of thymoquinone and to rebut the trivial deviance in retention time, each extract sample (NSEs) after treatment with doses of sodium azide was introduced together with standard pure thymoquinone solution. Pure thymoquinone standard curve with mean coefficient of determination (R^2^) = 0.99 and % RSD 7.84 was recorded. The extract obtained from the triplicate set of sodium azide treated calli were used in the analysis. The NS callus extract (NSE) yield was expressed as mg g^−1^ callus fresh weight, thymoquinone yield as µg g^−1^ callus fresh weight and thymoquinone content as mg g^−1^ callus extract weight.

### DPPH (2, 2-diphenyl-1-picrylhydrazyl) assay for evaluation of antioxidant activity

For free radical scavenging activity, DPPH method^53^ was used to estimate antioxidant activity. The reaction mixture comprised 0.3 mL DPPH (≥ 90% purity Merck, 300267) (0.5 mM in 100% ethanol) and 0.5 mL NSE and final volume was made 3.0 mL using ethanol. Briefly, 3.0 mL DPPH was added to 0.5 mL NSE and the reaction mixture was kept at room temperature for 60 min. The absorbance was taken at 517 nm. For blank 3.3 mL of ethanol and 0.5 mL NSE and for control 3.5 mL ethanol and 0.3 mL DPPH were used. The percent antioxidant activity was calculated according to the formula by Mensor et al.^54^.

$${\text{Percent antioxidant activity}} = 100-\left\{ {\left[ {\left( {{\text{Abs sample}}-{\text{Abs blank}}} \right) \times 100} \right]/{\text{Abs control}}} \right\}$$

### DNA damage inhibition assay

The antioxidant capacity of extracts from sodium azide treated NSEs was evaluated, employing the in vitro DNA damage inhibition assay protocol^55^. 0.025% calf thymus DNA (Ultra Pure ThermoFisher, 15633019) was prepared in 20 mM (pH 7.4) phosphate buffer saline (HiMedia, M1866). Thereafter, 1 mM thymoquinone and sodium azide treated NSEs were added in calf thymus DNA solution and then incubated at room temperature for about 15 min. Finally, the oxidative substances as mentioned in the protocol were added [i.e.,1μL of H_2_O_2_ (30 mM), 1μL ferric nitrate (AR grade; HiMedia, GRM1376) (20 mM), and 1μL ascorbic acid (AR grade; HiMedia, CMS1014) (100 mM)]. The whole reaction mixture was incubated for 1 h at 37 °C. The assay was performed in triplicates. DNA damage inhibition was visualized on agarose gel electrophoresis with the help of Gel Doc system (DNr Bio imaging system) and photographed. Inhibition percent of DNA damage was quantified at 260 nm wavelength (UV–Vis 1800 Schimadzu), using the following formula

$${\text{Percent Inhibition}} = \left[ {\left( {{\text{Absorbance control}}-{\text{Absorbance sample}}} \right) \div \left( {{\text{Absorbance control}}} \right)} \right] \times 100$$

$${\text{Sample}} = {\text{Calf DNA}} + {\text{Sodium azide treated NS callus extract}} + {\text{oxidative mixture}}$$

$${\text{Control}} = {\text{Calf DNA}} + 1\;{\text{mM Thymoquinone}} + {\text{oxidative mixture}}$$

### Experimental design and statistical analysis

The experiment was laid out as complete randomized design (CRD), taking sodium azide doses as treatment variable in three replicates. Various parameters were estimated in triplicates in each replicate and their mean value for each sodium azide treatment was used for statistical analysis employing one-way analysis of variation (ANOVA) model (tabulated below) with ANOVA values for different parameters as Supplementary Data (Supplementary Table S1). ‘F-test’ was adopted for test of significance at *p* value ≤ 0.05, using GraphPad Prism software version 5.01 (https://softadvice.informer.com/Graphpad_Prism_Version_5.01.html). If data was found significant, Tukey’s HSD value at *p* = 0.05 was computed to compare means of different parameters as significant effect of sodium azide treatments. Data for different parameters were represented as mean ± SD. Further, Pearson’s linear correlation coefficients (*p* < 0.05) among different pair-wise parameters was computed for ascertaining significant relationship.

**Table:** One way ANOVA (CRD) model.

Source of variation	df	SS	MSS	*F* value	*p* value
Treatment	6				
Error	14				
Total	20				

df = Degree of freedom, SS = Sums of square, MSS = Means of sums of square, values for treatment SS, MSS, *F* value and *p* value of each parameter is given in Supplementary Table S1.

### Ethic declaration

In our study all the protocols adhere with the ethical regulations on the usages of plants.

## Results and discussion

**Figure 1:**
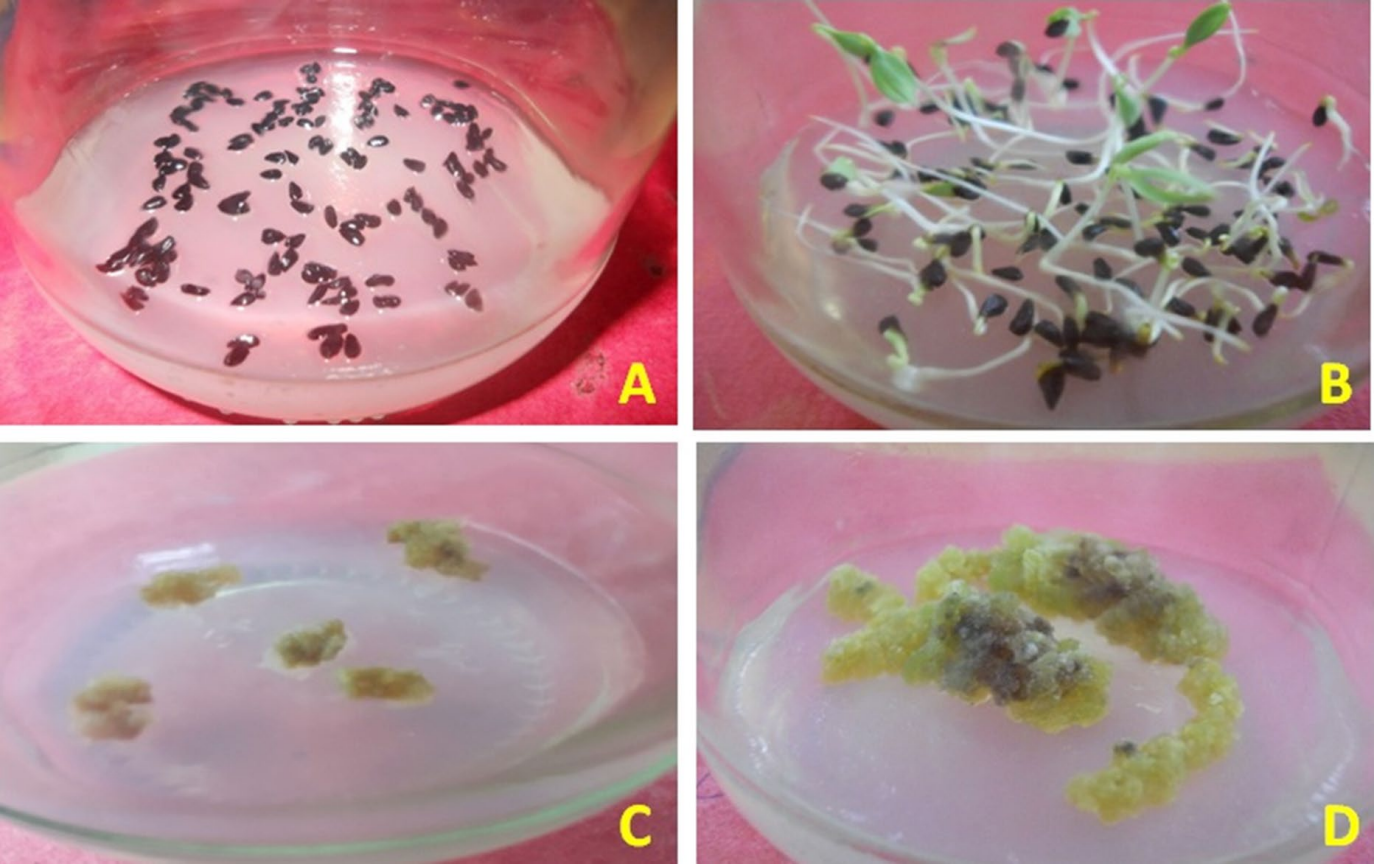
NS seed germination on MS medium and callus formation using plant tissue culture technique. (**A**) NS seeds on MS media; (**B**) NS seedlings; (**C**) Callus of NS in MS media supplemented 2,4-D; (**D**) Sodium azide treatment on NS Callus (n = 3).

The seeds of NS were germinated on Murashige and Skoog (MS) medium supplemented with 2,4-D (Fig. 1A,B) for the callus proliferation. The obtained calli (Fig. 1C) were transferred on MS medium with sodium azide as an elicitor (Fig. 1D).

**Figure 2:**
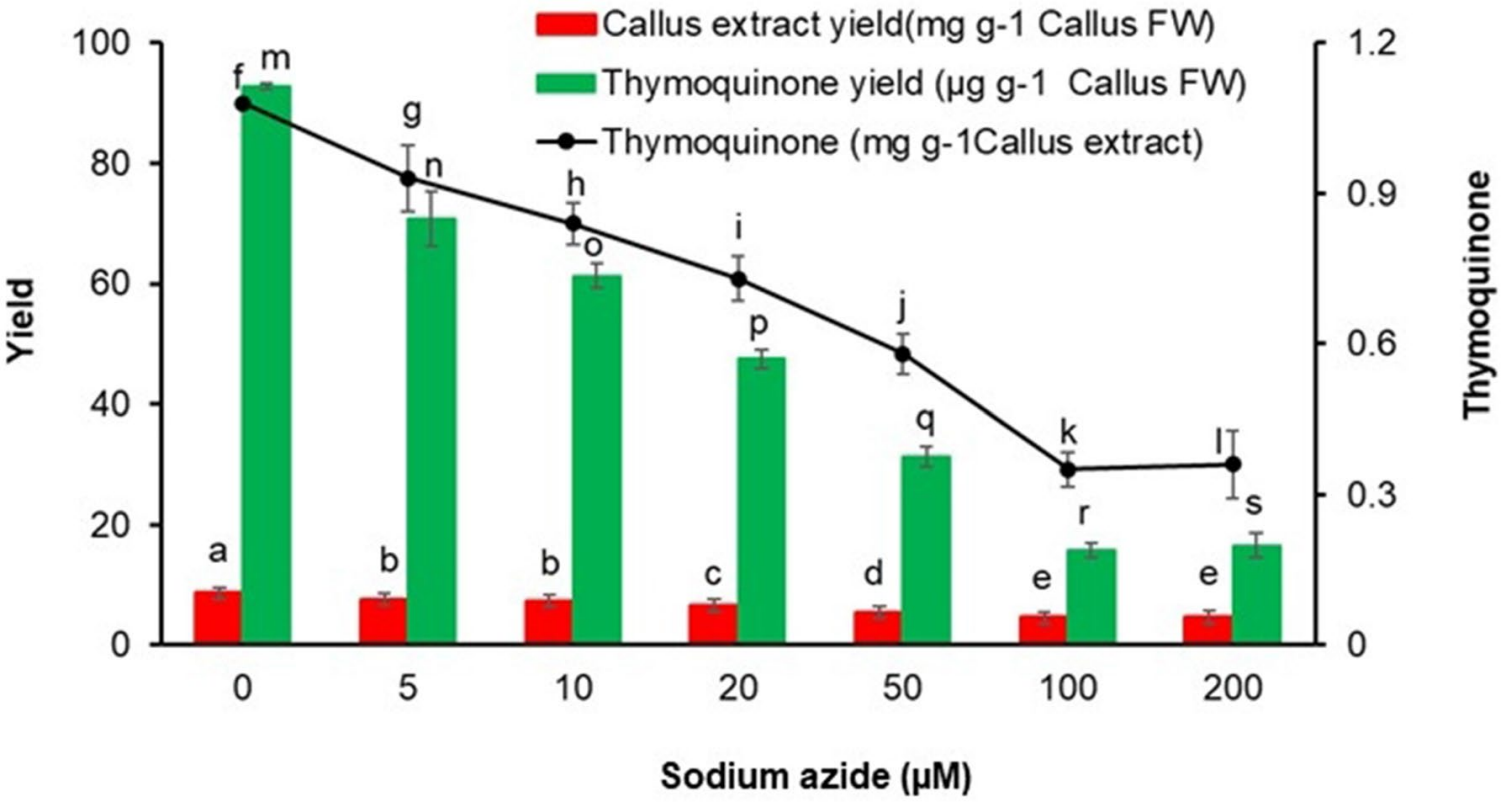
Graded doses of sodium azide significantly (*p* < 0.001) affect callus extract yield (mg g^−1^ callus FW) and thymoquinone yield (µg g^−1^ callus FW)/content (mg g^−1^ callus extract) in *Nigella sativa*, FW = Fresh weight. Vertical bars refer to ± standard deviation and histograms bearing the different alphabets in a series of data are significantly different from each other (Tukey’s HSD test).

**Figure 3:**
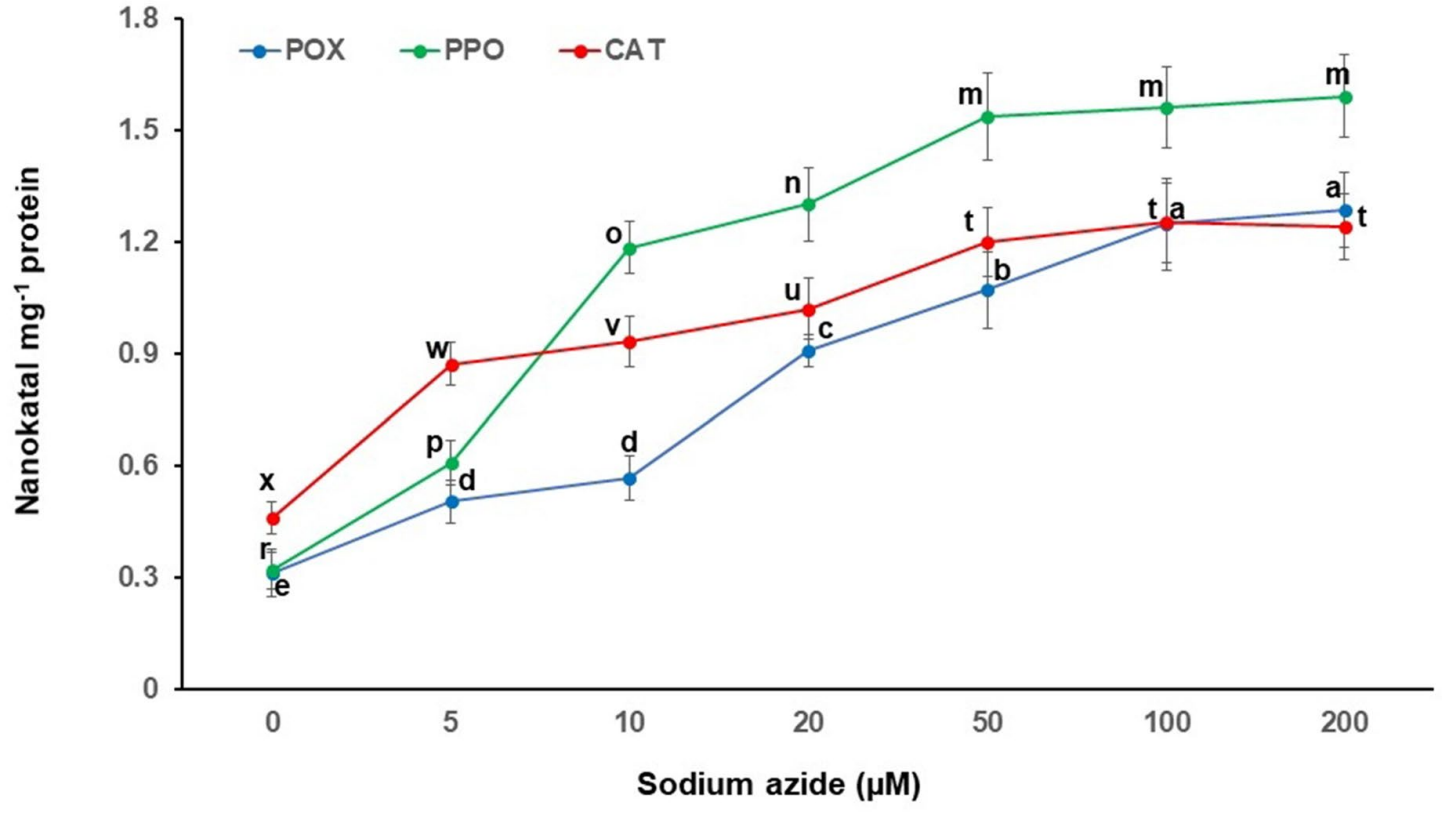
Graded doses of sodium azide significantly (*p* < 0.001) affect activities of POX, PPO and CAT in callus extract of *Nigella sativa*. Vertical bars refer to ± standard deviation and histograms bearing the different alphabets in a series of data are significantly different from each other (Tukey’s HSD test).

**Figure 4:**
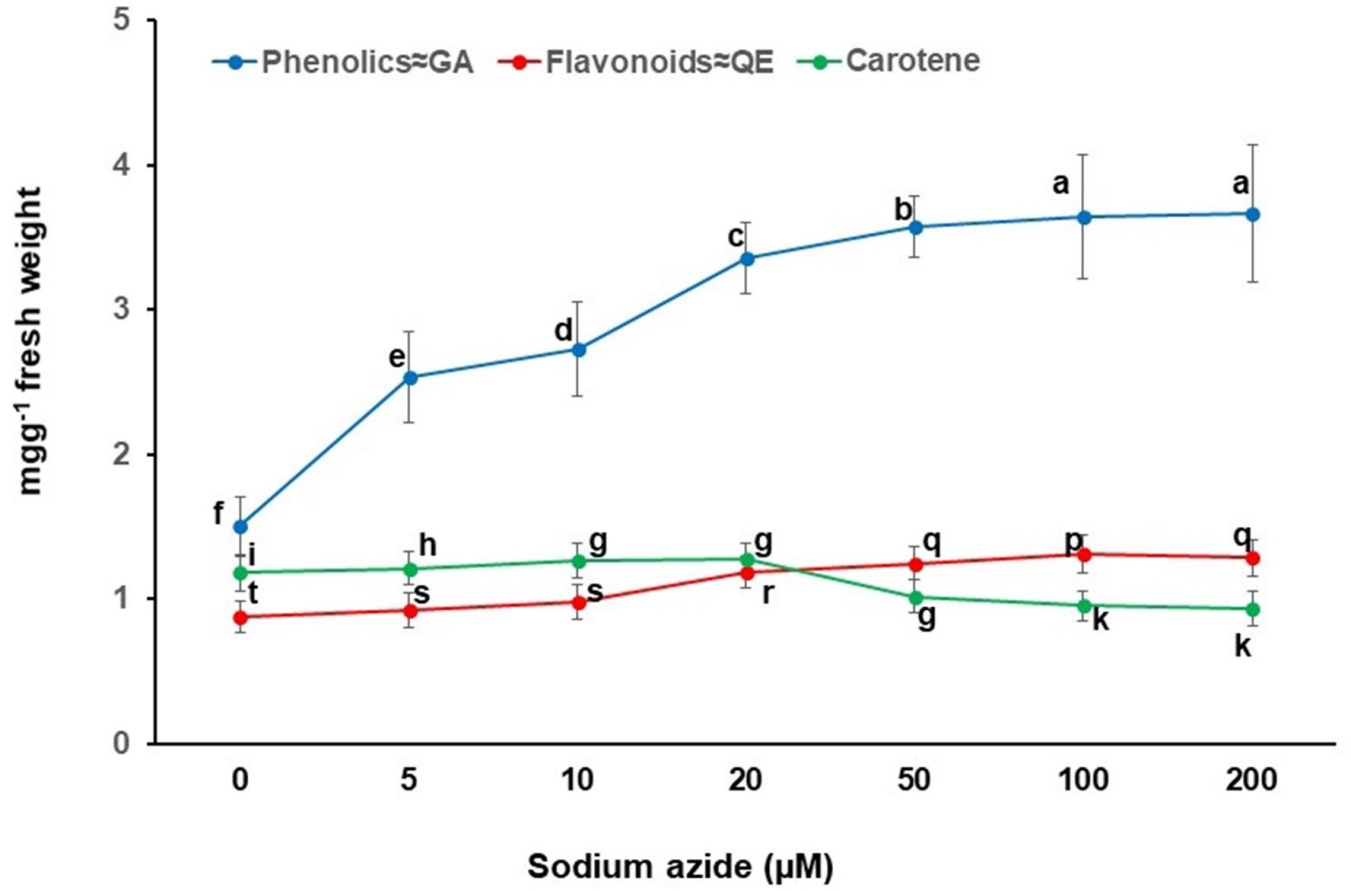
Graded doses of sodium azide significantly (*p* < 0.001) affect contents of phenolics, flavonoids and carotene in callus extract of *Nigella sativa*. Vertical bars refer to ± standard deviation and histograms bearing the different alphabets in a series of data are significantly different from each other (Tukey’s HSD test).

**Figure 5:**
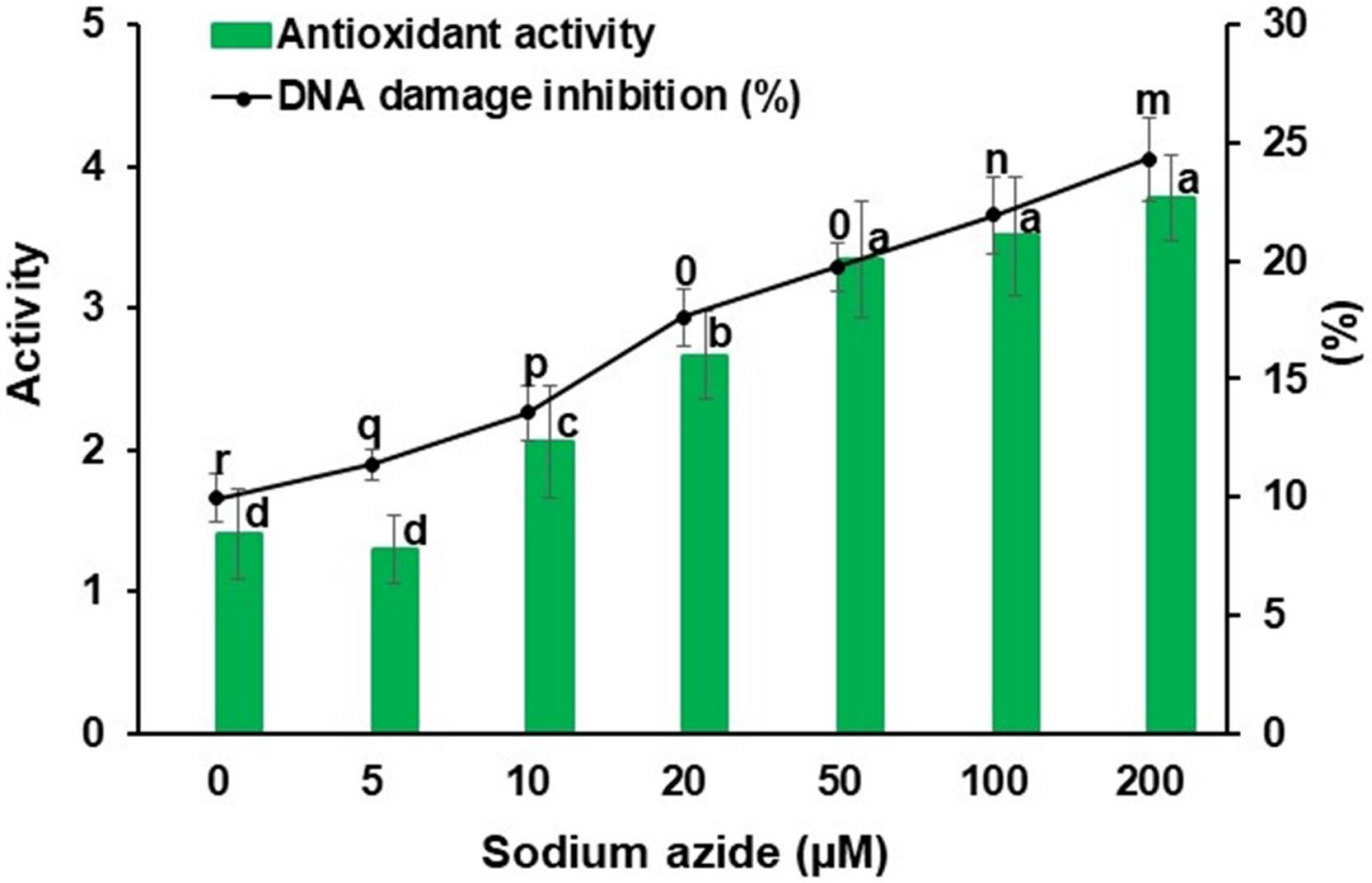
Graded doses of sodium azide significantly (*p* < 0.001) affect antioxidant activity of callus extract of *Nigella sativa* that also significantly (*p* < 0.001) corresponds to in vitro calf DNA damage inhibition (%). Vertical bars refer to ± standard deviation and histograms bearing the different alphabets in a series of data are significantly different from each other (Tukey’s HSD test).

The callus culture treated with sodium azide results in excess or deprived production of secondary metabolites^36,56^. A dose dependent sodium azide administration to NS, causing elevation in antioxidant system has been reported earlier^36^. However, it was observed that 100 µM concentration of sodium azide had elevated maximum antioxidant in NS^36^. NS callus administered with graded doses of sodium azide also experiences stress as evidenced by significant decrease in NSE yield and thymoquinone yield and content (Fig. 2) along with activation/expression of antioxidative scavenging system (Figs. 3, 4). For example, a rise in peroxidase, polyphenol oxidase and catalase activities were noted with respect to mounting sodium azide levels. The enzymatic antioxidants viz*.* peroxidase activity (1.286 ± 0.101 nanokatal mg^−1^ protein) as well as polyphenol oxidase activity (1.590 ± 0.110 nanokatal mg^−1^ protein) at 200 µM sodium azide and catalase activity (1.250 ± 0.105 nanokatal mg^−1^ protein) at 100 µM sodium azide were enhanced (Fig. 3). Similarly, sodium azide treatment increased total phenolics (3.666 ± 0.475 mg g^−1^ fresh weight) at 200 µM, total flavonoids (1.308 ± 0.082 mg g^−1^ fresh weight) and total carotenes (1.273 ± 0.066 mg g^−1^ fresh) at 20 µM (Fig. 4). The enhanced activity of total antioxidant (3.773 ± 0.302%) using DPPH was also observed at 200 µM (Fig. 4). The amplified production of ROS due to salt stress causes progressive oxidative damage which leads to suppressed growth and eventually death of cells/organisms^28^. Therefore, the cells activate metabolic tasks to scavenge ROS stress conditions. The task includes activation/elevation of antioxidant enzymes (peroxidase, polyphenol oxidase and catalase) and non-enzymatic secondary metabolites^57^. Our investigations also corroborate activity enhancement of antioxidant enzymes (Fig. 3) as well as non-enzymatic antioxidant metabolite contents, especially polyphenols (Fig. 4) due to sodium azide treatment to NS callus. Further, NS responds positively to sodium azide stress treatment in comparison to NaCl stress treatment (60 mM) that decreases phenolic acids and flavonoid content^58^. Previously, the salinity stress due to NaCl administration deprived the phenolic and flavonoid content in NS^58^. Our investigation also arrives at reiterating sodium azide as a stress initiator for the enhanced production of phenolic and flavonoids in NS. Presumably, sodium azide interrupts structural and functional components of NS callus cells that tend to adjust and repair cellular apparatus by eliciting antioxidant response such as production of secondary metabolites, phenolic acids and flavonoids^59^. Hence, sodium azide treated NS callus exhibited dose dependent rise in total antioxidant activity measured by DPPH assay in the present investigation (Fig. 5).

**Figure 6:**
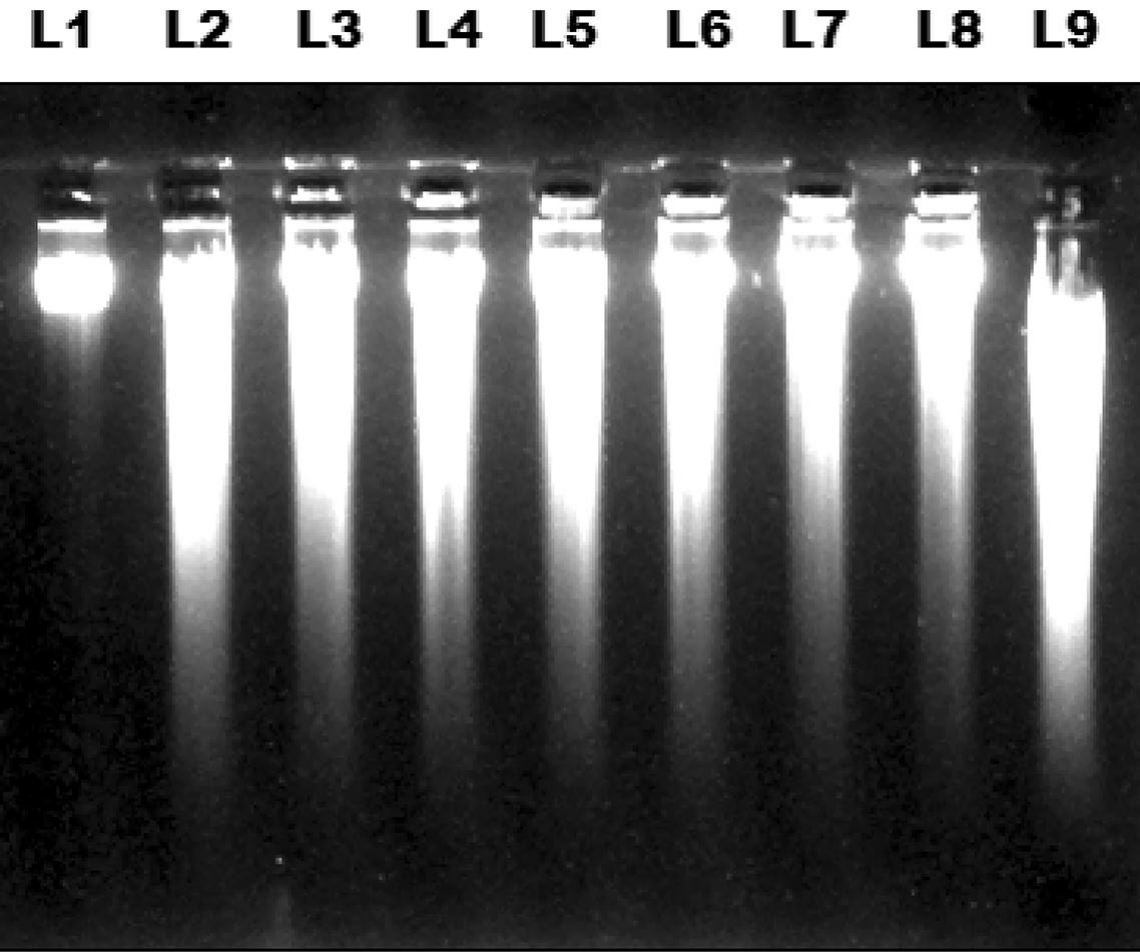
DNA damage inhibition on NSE after treatment with various concentrations of sodium azide. L1 Standard calf thymus DNA; L2, L3, L4, L5, L6, L7, L8 representing calf thymus DNA and NSEs after 0 (Control), 5, 10, 20, 50, 100, 200 µM treatment of sodium azide, respectively; L9 showing calf thymus DNA with 1 mM thymoquinone.

Further, the extracts of sodium azide treated NS callus and standard thymoquinone were investigated on calf thymus DNA damage inhibition activity. 200 µM sodium azide treatment maximally protected DNA damage (24.3 ± 1.7%) in comparison to the control treatment without sodium azide (Fig. 5). The probable reason for such results might be the formation of analogs of thymoquinone which are more potent antioxidants. This needs experimental verification, investigating synthesis of potent antioxidant thymquinone analogs as a result of sodium azide treatment to NS callus. Nevertheless, Fig. 6 vividly demonstrates calf thymus DNA protection against thymoquinone on receipt of extracts from sodium azide treated NS callus with diminished thymoquinone content. This indicates that NSE potential to protect calf DNA damage is independent of its thymoquinone content; but the same may be attributed to emergence of new peak with 5.3 min retention time. Thymoquinone at low doses (1–10 µg mL^−1^) reportedly protect DNA damage^60^. On the other hand, thymoquinone application in a dose-dependent manner initiates DNA damage in human glioblastoma cells^61^. Consequently, it needs to be emphasized that sodium azide treatment may have partly converted endogenous thymoquinone into its analogs in NS callus. These analogs might possess potent antioxidant activity that have protected DNA damage in the present investigation. Further, the process of DNA damage inhibition appears to have been synergistically augmented with the improvement in antioxidant activity of sodium azide treated NS callus extract. The argument gains weight from the previous finding which demonstrated the DNA damage protection by administration of *Crocus sativus* methanolic extract^62^. Hence, our finding comprises a preliminary novel report representing the in vitro inhibition of DNA damage ability of sodium azide treated NS callus.

**Figure 7:**
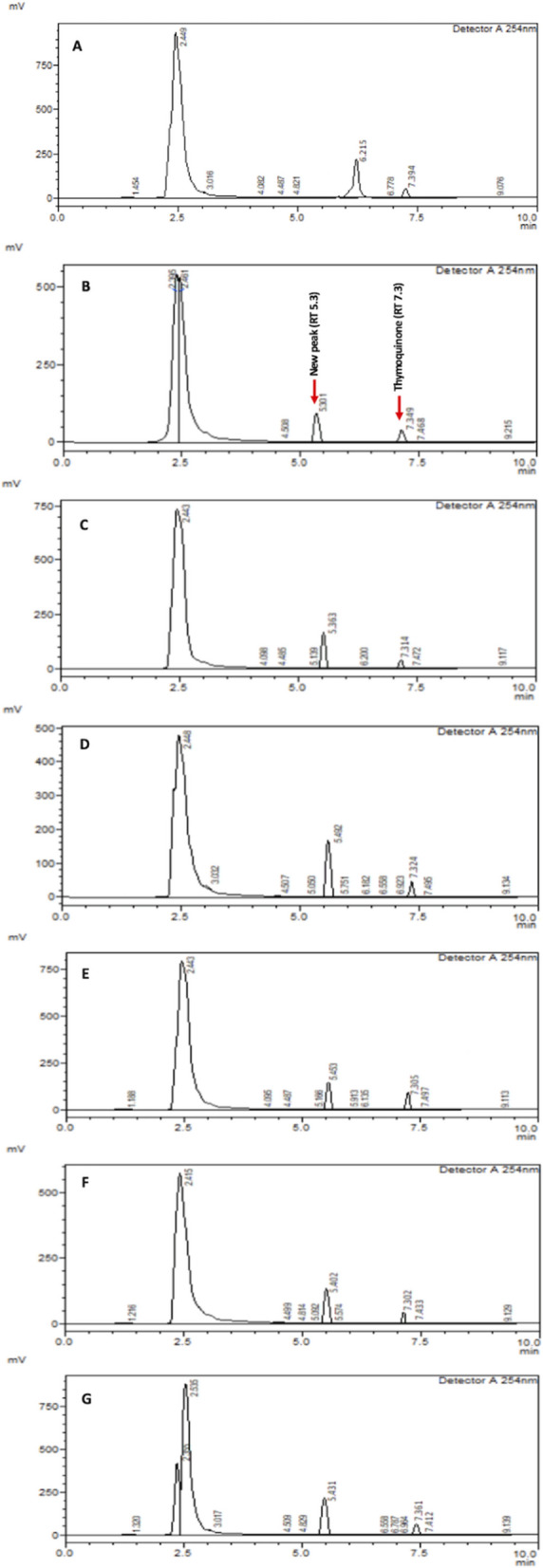
RP-HPLC histograms depict sodium azide dose-dependent new peak with retention time of 5.3 min along with thymoquinone peak (RT 7.3 min). (**A**) Control (0 µM sodium azide), (**B**) 5 µM sodium azide, (**C**) 10 µM sodium azide, (**D**) 20 µM sodium azide, (**E**) 50 µM sodium azide, (**F**) 100 µM sodium azide and (**G**) 200 µM sodium azide.

**Figure 8:**
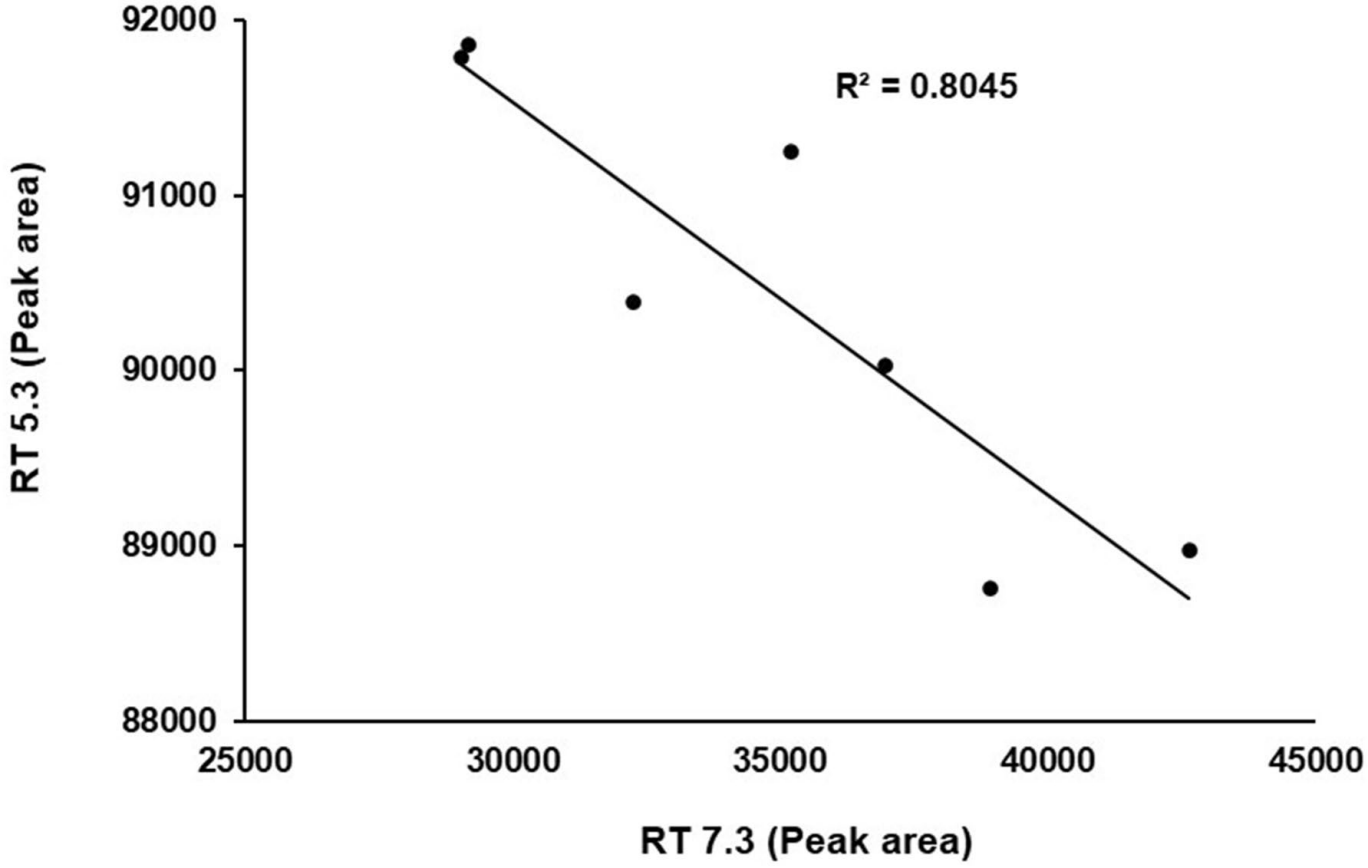
Significant negative correlation (*p* < 0.01) between peak area of RT 5.3 (New peak) and RT 7.3 (Thymoquinone).

**Figure 9:**
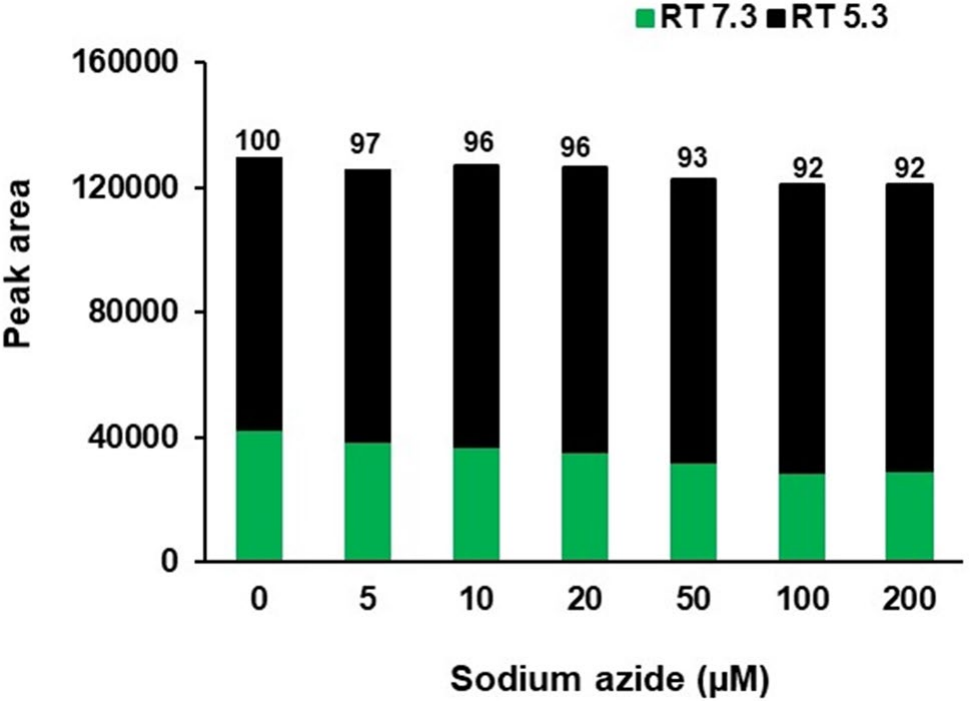
Sum of both peak areas (RT 5.3 and RT 7.3) across graded doses of sodium azide. Values on histograms denote comparison (%) with Control (100) and are not significantly different from each other.

It is interesting to note that the administration of sodium azide resulted in a dose-dependent decrease in thymoquinone yield and content of NS callus (Fig. 2). The plausible reason could be that the endogenous thymoquinone content may directly react with sodium azide for the synthesis of its analogs. As a result, NS callus decreased its thymoquinone content corresponding to increasing doses of sodium azide. It had been demonstrated that a chemical reaction occurs between thymoquinone and sodium azide to synthesize structural analog (3-amino-G-azido-2-methyl-5-isopropyl-1,4-benzoquinone) of the former^63–65^. Further, 2-azido-l,4-napthoquinone treated with azide ion and 2-chloroquinone yields azidoquinone^66^. In the given reaction, azide ions replace halogen ions in a nucleophilic displacement reaction, thereby yielding ≥ 85% azidoquinones^64^. Concomitantly, administration of sodium azide graded doses to NS callus produces an additional peak with retention time of 5.3 min whose area increases proportionately to sodium azide doses (Fig. 7) and inversely to thymoquinone peak area at retention time of 7.3 min (Fig. 8). However, the decrease in thymoquinone peak area is compensated by the area of new peak and cumulative area of both peaks remains almost constant across different doses of sodium azide (Fig. 9). Hence, our investigation provides an important lead for possible production of pharmaceutically efficient thymoquinone analogs in NS callus culture system by sodium azide treatment. The analogs may possess high antioxidant activity necessary for therapeutic usage^36^.

**Figure 10:**
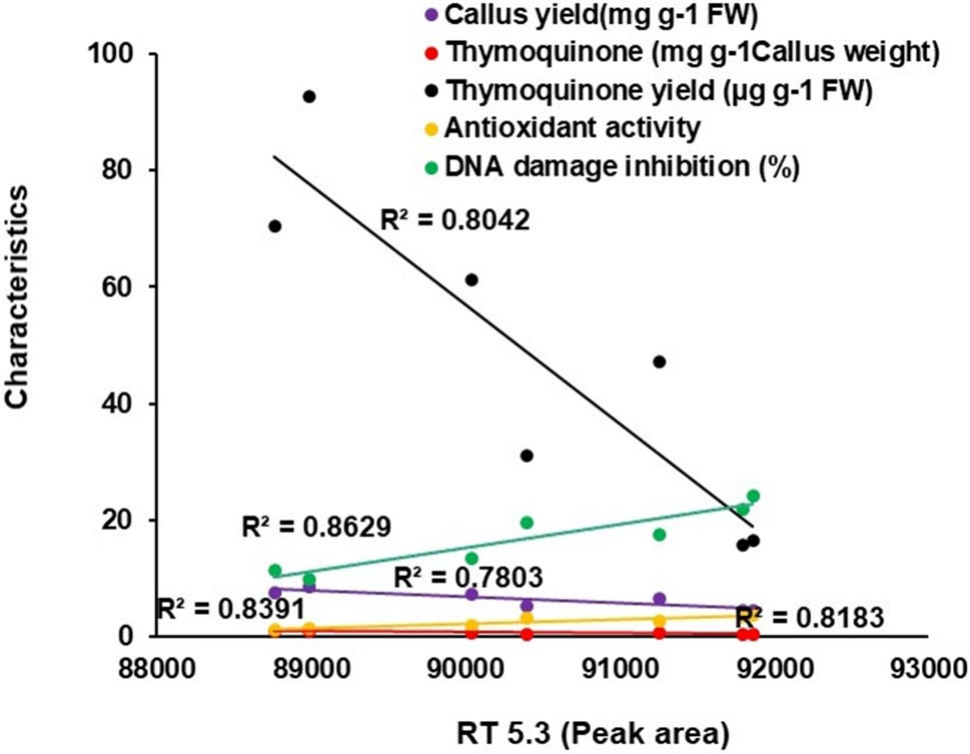
Peak area at 5.3 min retention time correlates negatively with callus yield (*p* < 0.05), thymoquinone yield and content (*p* < 0.01) and positively with antioxidant activity (*p* < 0.01) and DNA damage inhibition (*p* < 0.01).

Inconformity with the above findings, the peak area at 5.3 min retention time has significant negative correlation with callus yield, thymoquinone yield/content and significant positive correlation with antioxidant activity and DNA damage inhibition (Fig. 10). It explains very well that sodium azide treatment to NS callus induces abiotic stress on the one hand and converts endogenous thymoquinone to its more active analog on the other. As a result, callus yield decreases to compensate/repair damage caused by abiotic stress imposed by sodium azide treatment. For stress counter action, the NS callus has increased activities of antioxidant enzymes and secondary metabolites stress at the expense of its biomass. In the literature, Kumar et al.^67^ have also recorded similar loss of adventitious root biomass in *Gmelina arborea* due to administration of extreme acidic or basic pH stress conditions in order to adjust and repair cellular apparatus to cope with the adverse situation at the expense of constitutive metabolites. Nevertheless, the findings of our investigations are in agreement with the previous report of Herwibawa and Yafizham^68^ that pre-sowing seed treatment with 0–1.60 mM sodium azide of *Capsicum annum* L. alters various morphological and physiological parameters vis-à-vis decreased biomass. Iqbal et al.^36^ have also demonstrated that 0–100 µM sodium azide significantly affects extract yield in NS. A concomitant relationship between percent yield of the extract and chemical constituent exists in previous investigations^51,69^. Further, the new peak with 5.3 min retention time appears to be a thymoquinone analog that has been in vitro synthesized by direct action of sodium azide as discussed above. The analog has seemingly triggered antioxidant activity and conferred DNA damage inhibition to sodium azide treated NS callus. A previous report also suggests that sodium azide treatment affects antioxidants levels in NS, either by eliciting the level of secondary metabolites or by inducing conformational changes in its active phyto-constituents (mainly thymoquinone), thus elevating the antioxidant activity^36^ The antioxidant activity of thymoquinone is well known; however, its structural analogs are yet to be identified and explored for their elevated antioxidant activities. However, the investigation on structural analogs of thymoquinone could be an additional advantage resulting from the interaction of sodium azide with thymoquinone^63–65^.

## Conclusion and future perspectives

Thus, sodium azide treatment imposes stress conditions and vigorously affects secondary metabolites produced by NS. This includes enhancing antioxidant and DNA damage inhibition potentials in spite of dose-dependent decline in callus extract yield and thymoquinone yield/content. The obtained extract also inhibits DNA damage. This might be due to the elicitation of other secondary metabolites or the formation of thymoquinone analog (s) with more potent antioxidant activity. Consequently, the present study prompts some in-depth investigations on the in vitro sodium azide administration for obtaining desired deviation in metabolic pathways for enhanced levels of secondary metabolites or the formation of analogs of thymoquinone with more potent antioxidant activity within judicious time frame. To be specific, sodium azide administration to NS calli induces the emergence of a new metabolite with retention time of 5.3 min that correlates with antioxidant activity and calf DNA damage inhibition. Thus, sodium azide treated NS callus is recommended as a biological model for production of diverse efficient secondary metabolites of pharmaceutical novelty. Future research endeavors with the intervention of omics technology must focus to characterize the molecule and its potential uses in drug designing^70^. Broadly, genomics, transcriptomics and proteomics studies will reveal gene/genes responsible for thymoquinone biosynthesis and its analogs, which could be helpful on commercial scale in pharmaceutical and nutraceutical industries. The so produced bio-formulated compounds could be useful in treating various health ailments using natural process for the synthesis of active phytochemicals. Combination of proteo-genomics with peptide de-novo sequencing can identify potent genes and unknown post-transcriptional modifications^25,71^. Such in-vitro techniques for the production of enhanced level of phytochemicals meet the impending pharmaceutical challenges.

## Supplementary Information

Supplementary Figures.

Supplementary Table.

## Data Availability

The data used to support the findings of this study are available as supplementary data.
