# Supplementary material for: Nigella sativa callus treated with sodium azide exhibit augmented antioxidant activity and DNA damage inhibition
Source: Sci Rep. 2021 Jul 6;11:13954. doi: 10.1038/s41598-021-93370-x (PMC8260798; doi:10.1038/s41598-021-93370-x)
Supplement: Supplementary file 1 — Supplementary Figures. [file 41598_2021_93370_MOESM1_ESM.docx]

***Nigella sativa* callus treated with sodium azide exhibit augmented antioxidant activity and DNA damage inhibition**

Mohammed Shariq Iqbal, Zahra Iqbal, Abeer Hashem, Al-Bandari Fahad Al-Arjani, Elsayed Fathi Abd_Allah, Asif Jafri^6^, Shamim Akhtar Ansari, Mohammad Israil Ansari

Figure S1. Standard curve of gallic acid for total phenolics estimation.

Figure S2. Standard curve of quercetin for total flavonoids estimation.


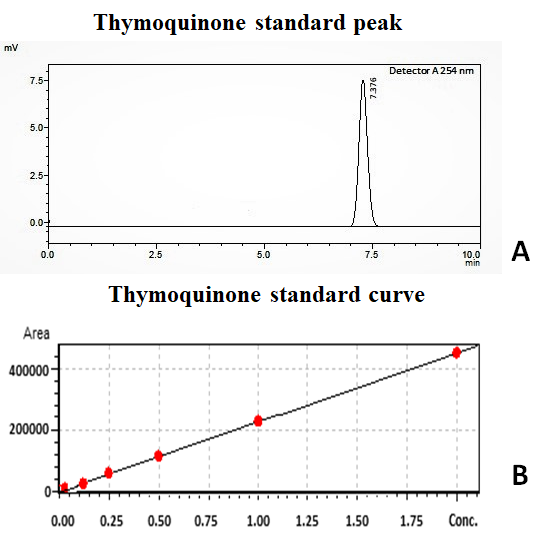


Figure S3. Determination of peak retention time of 7.376 min (A) and standard calibration curve ranging 0.0312 to 2.0 μg mL^-1^ for pure (99.0%) thymoquinone (B) with mean correlation coefficient of determination (R^2^) = 0.99 and % RSD =7.84.
