# Supplementary material for: Nigella sativa callus treated with sodium azide exhibit augmented antioxidant activity and DNA damage inhibition
Source: Sci Rep. 2021 Jul 6;11:13954. doi: 10.1038/s41598-021-93370-x (PMC8260798; doi:10.1038/s41598-021-93370-x)
Supplement: Supplementary file 2 — Supplementary Table. [file 41598_2021_93370_MOESM2_ESM.docx]

***Nigella sativa* callus treated with sodium azide exhibit augmented antioxidant activity and DNA damage inhibition**

Mohammed Shariq Iqbal, Zahra Iqbal, Abeer Hashem, Al-Bandari Fahad Al-Arjani, Elsayed Fathi Abd_Allah, Asif Jafri^6^, Shamim Akhtar Ansari, Mohammad Israil Ansari

Table S1. ANOVA values for various parameters.

| **S No.** | **Parameter** | **SS** | **MSS** | ***F value*** | ***p value*** |
| --- | --- | --- | --- | --- | --- |
| 1. | Callus extract yield | 4.44714 | 0.74119 | 29.93269 | 3.27E-07 |
| 2. | Thymoquinone | 0.13959 | 0.02327 | 105.6381 | 7.63E-11 |
|  | Thymoquinone yield | 9.70848 | 1.53E-03 | 245.4550 | 1.62E-02 |
| 3. | Antioxidant activity | 18.48209 | 3.08035 | 25.55195 | 8.88E-07 |
| 4. | DNA damage inhibition | 0.06823 | 0.00975 | 58.7628 | 3.15E-10 |
| 5. | Peroxidase (POX) | 2.66669 | 0.44444 | 63.9816 | 2.26E-09 |
| 6. | Polyphenol oxidase (PPO) | 4.55920 | 0.75987 | 91.57052 | 2.01E-10 |
| 7. | Catalase (CAT) | 1.41690 | 0.23615 | 37.30899 | 7.93E-08 |
| 8. | Phenolics | 11.54225 | 1.92371 | 17.61714 | 8.69E-06 |
|  | Flavonoid | 0.60610 | 0.10102 | 20.76472 | 3.21E-06 |
| 9. | Carotene | 0.39002 | 0.06500 | 9.325913 | 3.15E-04 |
| 11. | Peak area at RT 5.3 minutes | 2.89E+07 | 4812959 | 0.060627 | 0.998783 |
| 12. | Peak area at RT 7.3 minutes | 4.63E+08 | 77247983 | 2.659723 | 6.17E-02 |

SS=Sums of square, MSS= Means of sums of square, *F*= Treatment variance/ Error variance, *p*= Probability.
